# *Morinda citrifolia* L. leaf extract prevent weight gain in Sprague-Dawley rats fed a high fat diet

**DOI:** 10.1080/16546628.2017.1338919

**Published:** 2017-07-27

**Authors:** Najla Gooda Sahib Jambocus, Amin Ismail, Alfi Khatib, Fawzi Mahomoodally, Nazamid Saari, Muhammad Waseem Mumtaz, Azizah Abdul Hamid

**Affiliations:** ^a^ Faculty of Food Science and Technology, Universiti Putra Malaysia, Serdang, Malaysia; ^b^ Faculty of Medicine and Health Sciences, Universiti Putra Malaysia, Serdang, Malaysia; ^c^ Department of Pharmaceutical Chemistry, Kulliyyah of Pharmacy, International Islamic University Malaysia, Kuantan, Malaysia; ^d^ Department of Health Sciences, Faculty of Science, University of Mauritius, Reduit, Mauritius; ^e^ Halal Product Research Institute, Universiti Putra Malaysia, Serdang, Malaysia; ^f^ Department of Chemistry, University of Gujrat, Gujrat, Pakistan

**Keywords:** *Morinda citrifolia*, high fat diet, anti-obesity, pancreatic lipase, lipoprotein lipase, flavonoids

## Abstract

**Background**: *Morinda citrifolia* L. is widely used as a folk medicinal food plant to manage a panoply of diseases, though no concrete reports on its potential anti-obesity activity. This study aimed to evaluate the potential of *M. citrifolia* leaf extracts (MLE60) in the prevention of weight gain *in vivo* and establish its phytochemical profile.

**Design**: Male Sprague-Dawley rats were divided into groups based on a normal diet (ND) or high fat diet (HFD), with or without MLE60 supplementation (150 and 350 mg/kg body weight) and assessed for any reduction in weight gain. Plasma leptin, insulin, adiponectin, and ghrelin of all groups were determined. ^1^H NMR and LCMS methods were employed for phytochemical profiling of MLE60.

**Results**: The supplementation of MLE60 did not affect food intake indicating that appetite suppression might not be the main anti-obesity mechanism involved. In the treated groups, MLE60 prevented weight gain, most likely through an inhibition of pancreatic and lipoprotein activity with a positive influence on the lipid profiles and a reduction in LDL levels . MLE60 also attenuated visceral fat deposition in treated subjects with improvement in the plasma levels of obesity-linked factors . ^1^Spectral analysis showed the presence of several bioactive compounds with rutin being more predominant.

**Conclusion**: MLE60 shows promise as an anti-obesity agents and warrants further research.

## Introduction

There are four hundred million obese (body mass index (BMI) > 30 kg m^−^^2^) and 1.6 billion overweight (BMI = 25.0–29.9 kg m^−^^2^) people worldwide. Overweight and obese people have an increased risk factor for diabetes, cardiovascular diseases, and certain cancers [1]. Large cohort studies have also reported obesity to be independently related to increased oxidative stress in both men and women [2]. The positive correlation between increased level of C-reactive protein (CRP) and obesity confirm the fact that this disease is also a chronic inflammatory state, which can potentially lead to cardiovascular diseases [3]. Both developed and developing nations have not been spared by this public health burden. For instance, 16.3% and 6.3%, respectively, of Malaysian’s primary school children in an urban setting were found to be overweight and obese. The nutritional status of these Malaysian children was significantly related to blood pressure, history of breast-feeding, socio-economic factors, parents’ education level, and consumption of fast foods and sugary beverages [4]. One of the main causes of obesity is the consumption of diets high in fats. It is predicted that 51% of the American population will be obese by 2030, and if the obesity prevalence were to be maintained at its current levels, US$549.5 billion would be saved in terms of health care expenditure [5]. Effort to prevent and manage obesity is therefore primordial. Currently, there are limited drugs available for long-term treatment and management of obesity with proven efficacy and present drug development is still challenging due to the multifaceted nature of the disease [6]. There has been much effort to develop new anti-obesity agents from natural sources such as medicinal plants. Plant bioactive compounds, also termed phytochemicals, have been reported to possess anti-obesity effects through numerous mechanisms, such as inhibition of digestive and metabolic lipases, suppression of appetite, increase in thermogenesis, and inhibition of pre-adipocytes proliferation and differentiation [7,8]. Several reports on the anti-obesity effect of plant extracts in animal-based models of obesity have been published [9–12].

**Figure 1.**
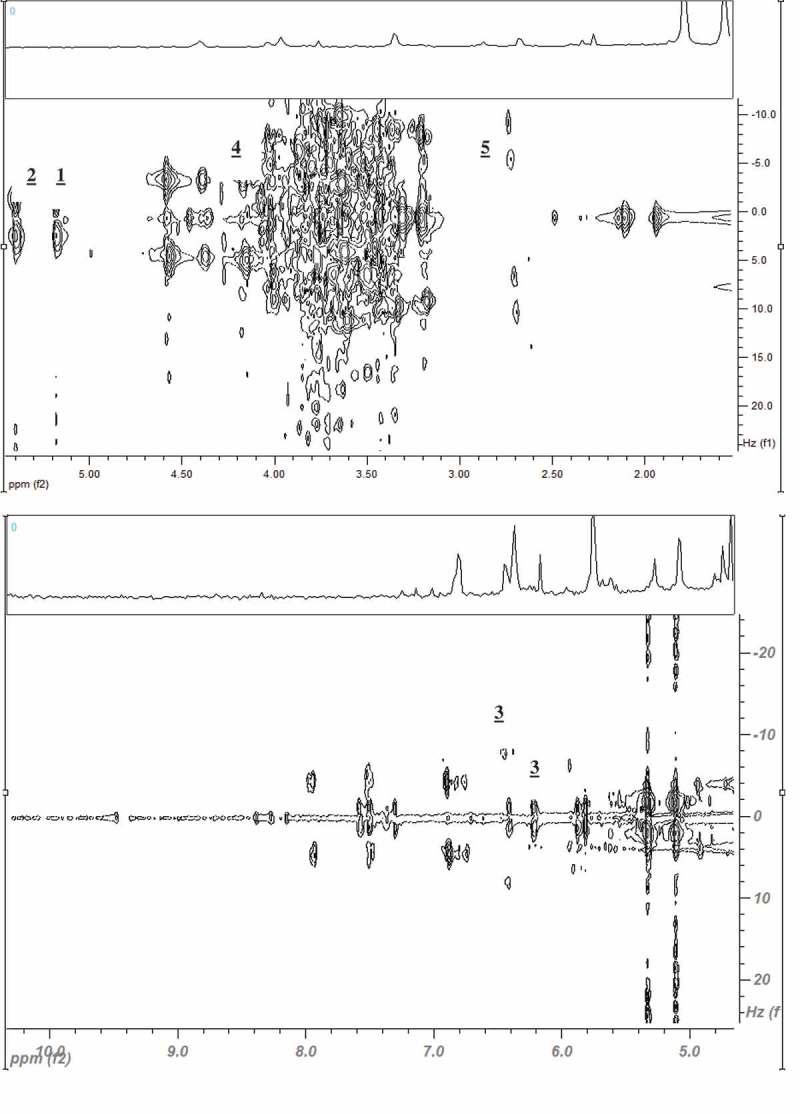
^1^H−^1^H *J*-resolved spectra of *M. citrifolia* mature leaves extracted with 60% of ethanol and 40% water (MLE60) in the region of δ 2.0 to 8.0 The observed signals are defined as [1] α-glucose, [2] sucrose, [3] kaempherol derivatives, [4] rutin derivatives, and [5] catechin derivatives.

**Figure 2.**
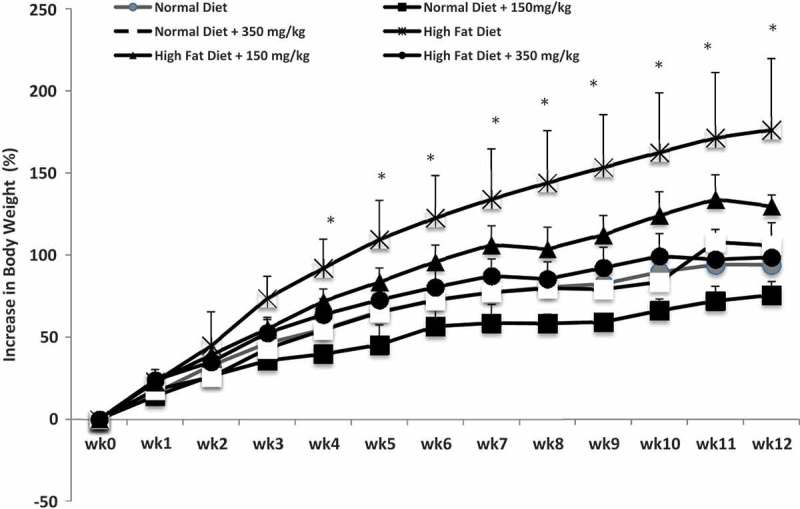
Percentage weight gain of rats fed a normal diet (ND) or a high fat diet (HFD) with our without the supplementation of 150 mg/kg body weight MLE60 or 350 mg/kg body weight MLE60. Values with * indicate significant difference (*p* < 0.05) in weight gain between different groups, during the same week of the experiment. All statistical analysis was carried out using one way ANOVA MINITAB version 14.

The potential anti-obesity effect of *Morinda citrifolia* L. fruit extracts *in vitro* has been previously reported [7,13]. The leaves, fruits, and roots of *M. citrifolia* L. are widely used in folk medicine in Southeast Asia, Polynesia, Tahiti, Australia, and Hawaii [14] and are associated with many health-promoting properties. It is believed to protect against inflammatory diseases such as diabetes and against oxidative stress [15]. Leaves of *M. citrifolia* L. are most commonly used in tablet forms as tea [16]. However, there is still a dearth of scientific validation of the potential anti-obesity effect of *M. citrifolia* L. leaves *in vivo*.

The present study was geared towards evaluating the potential of *M. citrifolia* leaf extracts (MLE60) in the prevention of weight gain *in vivo* and establish its phytochemical profile. Male Sprague-Dawley rats were divided into groups based on a normal diet (ND) or high fat diet (HFD), with or without two different doses (150 and 350 mg/kg body weight) of MLE60 supplementation and assessed for the prevention of weight gain. A number of plasma biomarkers (blood glucose level, total cholesterol (TC), low-density lipoprotein (LDL), high-density lipoprotein (HDL), triglycerides (TG), urea nitrogen, creatinine, γ-glutamyltransferase (GTT), alanine aminotransferase (ALT), aspartate aminotransferase (AST), and alkaline phosphatase activity (ALP)) were measured in the treated and untreated HFD groups and compared with rats fed on the ND. Plasma leptin, insulin, adiponectin, and ghrelin were determined using standard methods. Total phenolic content (TPC), DPPH radical scavenging, and pancreatic and lipoprotein lipase activity were determined. ^1^H nuclear magnetic resonance (NMR) and liquid chromatography mass spectroscopy (LCMS) methods were employed for phytochemical profiling of MLE60.

## Materials and methods

### Collection and extraction of raw materials

Fresh mature leaves of *M. citrifolia* were collected from Bukit Expo, Universiti Putra Malaysia. A voucher specimen (SK 2197/13) was deposited at the herbarium, Institute of Bioscience, Universiti Putra Malaysia, and the species was confirmed as *M. citrifolia* L. by a local botanist. Leaves were collected consistently in the morning, from 10 to 11 o’clock, on sunny days, from five representative trees. The leaves were cleaned and treated with liquid nitrogen for immediate quenching of metabolites and were stored at −80°C for 24 h. The frozen leaves were then lyophilised under vacuum, until constant weight (48–72 h), ground, and stored at −80°C until further use. Powdered leaves (10 g) were extracted with 100%–60% ethanol for 72 h at room temperature. The solvent was filtered out every 24 h and the pooled filtrate was evaporated using a rotary evaporator until a viscous extract was obtained. Crude extracts were lyophilised under vacuum until constant weight (36–48 h) and kept at –80°C until further use. They were defined as MLE100, MLE80, MLE60, MLE50, and MLE40 based on the percentage of ethanol used in the extractions.

### ^1^H NMR measurement and data analysis

MLE60 (25 mg) were dissolved in 0.375 mL of CH_3_OH-*d*_4_ without any internal standard and 0.375 mL of KH_2_PO_4_ buffer in D_2_O (pH 6 adjusted with NaOD) containing 0.1 (w/w) TSP. The mixture was vortexed for 1 min and ultrasonicated for 10 min at room temperature. The solution was then centrifuged at 13,000*g* for l0 min and 600 μL of the supernatant was transferred to a 5 mm NMR tube and subjected to ^1^H NMR analysis [17]. Spectra were acquired at 27°C on a Varian Unity INOVA 500 MHz spectrometer (Varian Inc, CA), with a frequency of 499.887 MHz. For each sample, 64 scans were recorded with an acquisition time of 193 s, pulse width of 3.75 μs and relaxation delay of 1.0 s. Additional two-dimensional (2D) ^1^H–^1^H *J* resolution was performed to aid in the identification of certain metabolites.

### TPC

TPC was quantified using the Folin–Ciocalteau method, as proposed by Singleton at al. [18]. Methanolic extracts (0.5 mL), 0.5 mL Folin–Ciocalteau, and 10 mL of 7% of sodium carbonate were allowed to react for 1 h. The resulting blue complex was read at 725 nm (Shimadzu UV visible Spectrophotometer, UV-1650 PC, Japan).

### DPPH radical scavenging activity

The antioxidant activity of the extracts was determined on their ability to scavenge DPPH radicals, based on a modified method of Brand-Williams et al. [19]. Different concentrations of MLE60 was dissolved (0.25 mL) in methanol were mixed with 1.75 mL of 6 × 10^−^^5^ mol of DPPH solution in a 12-well plate and allowed to stand for 30 min at room temperature, in the dark. Pure methanol was used as baseline control. The absorbance value was read at 515 nm using an Elisa plate reader (Biotek, EL 800). Antioxidant activity was reported as IC_50_, define by the concentration of samples required (mg/mL) to scavenge 50% of the free radicals. Butylated hydroxyanisole (BHA) and α tocopherol were used as positive controls for synthetic and natural antioxidant respectively.

### Pancreatic and lipoprotein lipase activity

Pancreatic and lipoprotein lipase activity was carried out based on previous methods, as described elsewhere [7,13], both titrimetric methods based on the quantification of free fatty acids released in a substrate, lipase and extract system as compared to a substrate and lipase system only.

### LCMS analysis

Dried extract (10 mg) was suspended in 1 mL methanol and filtered through a PTFE filter (pore size 0.22 µm). The analysis was carried out on a LTQ Trap mass spectrometer (Thermo Scientific) with U-HPLC system (Accela). Spectral *m/z* from 100–1000 was recorded and the MS^n^ fragmentation was carried out with 35–40% collision energy. Electrospray ionisation conditions were as follows: source accelerating voltage 4.0 kV; capillary temperature, 275°C; sheath gas flow, 40 arb; auxiliary gas, 20 arb. A Hypersil Gold RP C_18_ column (2.1 mm ID × 100 mm, 3 µm) was used for elution. Aqueous formic acid (0.1%) and acetonitrile were used as eluting solvents. A flow rate of 200 µL/min and an injection volume of 5 µL were used.

### HPLC analysis of MLE60

Dried extract (10 mg) was dissolved in 1 mL methanol and filtered through a 0.45 μm (Whatman) nylon membrane filter prior to injection into HPLC system. Analysis was run in triplicates. MLE60 was profiled for commonly present flavonoids (rutin, kaempherol, quercetin, myricetin, fisetin, hesperitin, quercitrin, naringin, and genistein. Major identified compounds were further quantified using a standard curve. Standards (60–140 ppm) were dissolved in methanol for quantification. The HPLC system consisted of a Waters Delta 600, with a 600 controller and photodiode array detector (Waters 996). The column used was a Phenomenex-Luna (5 µm) PFP[2] column (4.6 mm ID × 250 mm) and two eluting solvents were used. Solvent A was 0.1% aqueous formic acid and solvent B was acetonitrile. A flow rate of 1.0 mL/min and an injection volume of 10 μL were used. Retention times, peak areas, and UV spectra of major compounds were further analysed.

### Animal experiment

Male Sprague-Dawley rats (5 weeks old) were purchased from Sapphire Enterprise, Malaysia and acclimatised for 10 days under standard laboratory conditions (12 h light/dark cycle, 55–60% relative humidity, 23–25°C). After acclimatisation, rats were randomly divided into two groups based on assigned diets: standard rat chow (Gold Coin, Malaysia) and an HFD (MP Diets, USA). The composition of the both diets is given in Table 1. Animals in both groups were further subdivided into the following groups (*n* = 6), based on supplementation or non-supplementation with MLE60:

- ND = normal diet only
- ND+150 = normal diet + 150 mg /kg body weight of MLE60
- ND+350 = normal diet + 350 mg/kg body weight MLE60
- HFD = high fat diet only
- HFD+150 = high fat diet + 150 mg/kg body weight MLE60
- HFD+350 = high fat diet + 350 mg/kg body weight MLE60

**Table 1.** Composition of experimental diets.

Normal diet (ND) Standard rat chow Gold Coin, Malaysia(3.27kcal/g)		High fat diet (HFD) High saturated fat diet MP Diets, USA(Cat Num 960,242) (4.39 kcal/g)	
Ingredients	Percentage/amount	Ingredients	Percentage/amount
Crude protein	21–23%	Casein purified	20%
Crude fibre	5.0%	DL-Methionine	0.3%
Crude fat	3.0%	Sucrose	30.58%
Moisture	3.0%	Corn starch	20%
Calcium	0.8–1.2%	Coconut oil hydrogenated	20%
Phosphorus	0.8–1.2%	Alphacel-non-nutritive bulk	5%
Nitrogen-free extract	49.0%	dl-a-Tocopherol powder (250 IU/g)	0.12%
Vitamin A	10 MIU	AIN-76 Mineral Mix	4.0%
Vitamin D_3_	2.5 MIU	MP Vitamin Diet Fortification mixture 1.2X normalamount	
Vitamin E	15 g		

AIN-76 mineral mix contains the following (per 1000 g): calcium phosphate dibasic 500 g, sodium chloride 74 g, potassium citrate monohydrate 220 g, potassium sulphate 52 g, magnesium oxide 24 g, magnesium carbonate 3.5 g, ferric citrate 6 g, zinc carbonate 1.6 g, cupric carbonate 0.3 g, potassium iodate 0.01 g, sodium selenite 0.01 g, chromium potassium sulphate 0.55 g, sucrose 118 g.

### Administration of MLE 60

Animals were allowed their respective diets *ad libitum* and appropriate dosage of MLE60 was given through gastric intubation using a force-feeding needle. Volume of extracts given per day did not exceed 3.0 mL. Control groups received the carrier vehicle (0.03% CMC) through gastric intubation. Rats were allowed a sedentary lifestyle, without any specific physical activity regime for the course of the experiment.

### Measurement of body weight, food intake and collection of faeces

Body weight of rats was measured weekly using a weighing balance. The % weight gain was calculated as: (body weight on specific week (g) − initial body weight)/initial body weight ×100.

Food intake was measured once a week over 24 h based on the weight of leftover feed out of 100 g given. Faecal samples were recorded at the middle (week 6) and at the final stage (week 12) of the study using metabolic cages over a period of 18 h.

### Sacrifice of animals

After 12 weeks of treatment, animals were weighed and sacrificed by cardiac puncture under an anaesthetic effect (xylazine + ketamine). Rats were deprived of food for 12 h prior to sacrifice. Serum and plasma samples were separated at 1500*g* for 15 m and stored at −80°C for further analysis. Organs (liver, kidney, lung, heart, testis, and adipose tissue) were weighed, rinsed with saline, and fixed in 10% formalin for histopathological analysis. All animals were handled according to the international principles of the use and handling of experimental animals (US National Institute of Health, 1985) and all the protocols were approved by the Animal House and Use Committee of the Faculty of Medicine and Health Sciences, Universiti Putra Malaysia (Approval no: UPM/FPSK/PADS/BR.UUH/00462).

### Blood biochemistry

Rats were fasted for 12 h before blood glucose measurement. Food was removed from the food dispenser and bedding of the cages was changed to avoid coprophagy. Blood from the tail vein was measured for glucose using the OneTouch Basic glucose monitor (Lifescan) and reported as mmol/L. Blood glucose level was measured during the study and prior to sacrifice. TC, LDL, HDL, TG, urea nitrogen, creatinine, GGT, ALT, AST, and ALP activity were measured on a Roche/Hitachi cobac C system and results were expressed in U/L (Roche Diagnostics GmbH, Sandhofer, Strasse, Mannheim), following manufacturers’ protocols. Plasma leptin, insulin, adiponectin, and ghrelin were determined using enzyme-linked immunoabsorbent assay (ELISA) kits following manufacturer’s instruction (leptin: RayBio® Rat Leptin ELISA Kit, Cat # ELR-Leptin-001, Norcross, GA, USA; insulin: Mercodia Rat Insulin ELISA, Uppsala, Sweden; adiponectin: Assay-Max Rat Adiponectin ELISA kit, Cat# ERA2500-1; ghrelin: RayBio® Rat Ghrelin ELISA kit, Cat# EIA-GHR-1, Norcross, GA, USA).

### Determination of faecal fat content

Faecal lipid content was determined according to a modified method of Tsujita et al. (2006, 20). Faces were collected at the middle (week 6) and end (week 12) of the experiment. Faces (0.5 g) were soaked in 2 mL of deionised water for 24 h at 4°C, followed by homogenisation by vortexing at high speed for 60 seconds. Lipids were extracted with 7.5 mL of methanol: chloroform (2:1, v: v) and shaken for 30 m, followed by addition of 2.5 mL of deionised water and 2.5 mL of chloroform and further shaking for 30 m. Mixture was then centrifuged at 2000 *g* for 15 min and the lipophilic layer from the extraction was collected and dried under vacuum. Total fat content was weighed using a laboratory balance.

### Statistical analysis

Data is expressed as mean ± standard deviation (SD). Difference between groups was determined by one-way analysis of variance (ANOVA, MINITAB, version 14.0). Values were considered to be significantly different at the level of *p* < 0.05. In the analysis of faecal fat content (week 6 and week 12) and body weights (before and after treatment), significance was further confirmed with one sample t test.

## Results and discussion

### Bioactive content and bioactivity of different extracts of M. citrifolia leaf

The extracts were assessed for their TPC, DPPH scavenging potential and inhibitory effect on pancreatic and lipoprotein lipase as a preliminary evaluation (Table 2). Mature leaves of *M. citrifolia* were extracted using different concentration of ethanol:water (100–60:0–40). Leaves extracted with 60–40% ethanol had higher TPC and DPPH scavenging activity whereas leaves extracted with 100–80% ethanol had higher content of TFC (Table 2). Different solvent systems have been used to extract polyphenols from plants, with varying results. Polar solvents are most suitable for extraction of polyphenols from a plant matrix, with ethanol, methanol, acetone, and ethyl acetate being the usual solvent of choice [21]. A bi-solvent system has been proposed to be more efficient than a single pure solvent. Sultana et al. previously showed that in general a higher extraction yield, phenolic content, and antioxidant activity was obtained in an aqueous based solvent system as compared to a pure solvent system [22]. More specific to *M. citrifolia*, a binary solvent extraction system (40% ethanol and 60% water) for 80 min at 65°C had the highest recovery of total phenolic and flavonoids compounds, showing a positive correlation with the antioxidant capacity [23]. Results of this study is in accordance to reports found in literature which support that lower concentrations of ethanol (50%) manages to extract more polyphenols and show more potent antioxidant activity as compared that of pure ethanol.

**Table 2.** TPC, DPPH scavenging activity, and PL and LPL inhibiting activity of extracts of *M. citrifolia* leaves of different maturity.

Samples	TPC (g GA equivalent/ 100 g extract)	DPPH scavenging activity (IC_50_ (mg/mL)	Inhibition of PL activity at 0.625 mg/mL (%)	Inhibition of LPL activityat 1 mg/mL (%)
Mature (100:0)	8.98 ± 0.233^b^	1.05 ± 0.320^c^	6.00 ± 1.13^c^	11.80 ± 0.11^bc^
Mature (80:20)	8.45 ± 1.230^b^	1.00 ± 0.201^c^	8.95 ± 2.47^bc^	8.93 ± 1.88^b^
Mature (60:40)	11.85 ± 0.134^a^	0.86 ± 0.055^b^	12.75 ± 2.05^ab^	15.18 ± 1.53^a^
Mature (50:50)	11.29 ± 1.153^ab^	0.79 ± 0.104^b^	9.50 ± 0.42^bc^	14.44 ± 0.41^a^
Mature (40:60)	10.11 ± 0.103^ab^	0.90 ± 0.096^b^	6.70 ± 0.99^c^	13.46 ± 1.80^a^
BHA	NA	0.02 ± 0.001^a^	NA	NA
Tocopherol	NA	0.04 ± 0.001^a^	NA	NA
Orlistat	NA	NA	18.23 ± 2.51^a^	NA
Epicatechin	NA	NA	3.30 ± 0.42^d^	11.89 ± 1.37^ab^

Different small letters denote significant difference between the extracts for the same activity. Mature (100:0) indicate mature leaves extracted with 100% (v:v) ethanol:water; Mature (80:20) indicates 80:20 (v:v) ethanol:water; etc. Statistical analysis has been carried out using MINITAB 14 and significance value set at *p* < 0.05. All analysis was repeated three times, with triplicate readings.

An ethanol water system is commonly used for extraction as not only it can dissolve a wide range of polyphenols, but also is accepted for human consumption [24]. The different extracts of the mature leaves were also assessed for their inhibitory activity on pancreatic and lipoprotein lipase *in vitro*. At a low concentration of 0.625 mg/mL, mature leaves extracted with 60% ethanol inhibited pancreatic lipase (PL) by 12.75 ± 2.05% as compared to pure epicatechin, which showed a mild inhibition of 3.30 ± 0.42% (Table 2). At this concentration, the inhibition was not significantly different from that of Orlistat®, which was used as positive control. The extracts also showed inhibition of lipoprotein lipase activity. At 1 mg/mL, mature *M. citrifolia* leaves extracted with lower concentration of ethanol (60–50%) had the highest percentage inhibition with 15.17 ± 1.53 to 13.46 ± 1.80, which was not significantly different from the inhibition achieved by pure epicatechin at 11.89 ± 1.37%. Grape seed extract and peanut seed extract (0.1 mg/mL) showed 22% and 39%, respectively, inhibition of human PL activity as measured with the Lipase PS kit [25]. Four hundred plants were screened for their anti-obesity activity by assessing their effect on PL *in vitro*. Four species, including *Rubifructus, Cornifructus, Salicis radicis cortex*, and *Geranium nepalense*, were the most potent lipase inhibitors, exhibiting at least 30% inhibition. The anti-obesity effect of the extracts was further confirmed by their ability to reduce lipid uptake in 3T3-L1 adipocytes [26].

Very few studies attempted to study the inhibition of LPL by plants extracts. Moreno et al. assessed the effect of grape seed extract and peanut nutshell extract *in vitro*, showing an average inhibition of 30% at 1 mg/mL [25]. The inhibition potential was attributed to a synergistic effect of various plant bioactives, especially polyphenols. An ethanolic extract of *M. citrifolia* fruit extract was reported to inhibit LPL activity by 21.5 ± 2.3% [13], which is higher than the inhibition achieved by leaf extracts shown in this study. Extracts with lower concentration of ethanol (60–40%) showed significantly higher inhibition (*p* < 0.05) compared to leaves extracted with pure ethanol.

Based on their TPC, DPPH scavenging activity, and lipase inhibiting potential, mature leaf extracted with 60% ethanol, defined as MLE60, was selected for further analysis using ^1^H NMR and 2D ^1^H–^1^H NMR and LCMS/HPLC for identification and quantification of metabolites, respectively.

### ^1^H NMR spectra and assignments of compounds in MLE60

Certain metabolites were discernible in MLE60 as analysed by ^1^H NMR. Due to overlapping of peaks, *J*- resolve and heteronuclear multiple-bond correlation spectroscopy (HMBC) were used to gather more information on signal splitting and the coupling constant for the confirmation of compounds with those reported in literature (Figure 1). A list of identified compounds is given in Table 3. Among the important compounds are catechin, kaempherol and rutin, which are three flavonoids reported to have anti-obesity effects. These compounds together with other flavonoids like quercetin have been identified in the leaves of *M. citrifolia* previously [27]. Rutin has been reported to have anti-obesity effect in many *in vitro* and *in vivo* studies [28]. LCMS identification and HPLC quantification of commonly present flavonoids in plants in MLE60 revealed the presence of rutin as the predominant compound among the flavonoids tested. MLE60 contained 16.46 ± 0.91 mg of rutin/g of dried plant extract. In relation to obesity, *in vivo*, intervention with 50 and 100 mg rutin/kg body weight resulted in prevention of weight gain in HFD-induced obese *Wistar* rats [29]. Based on the preliminary findings, MLE60 was assessed for prevention of HFD-induced obesity in Sprague- Dawley rats.

### Effect of MLE60 on body weight, food intake and faecal fat excretion

Excessive calorie intake contributes to the development of obesity. The preventive effect of MLE60 on weight gain, appetite and faecal fat excretion in lean male Sprague-Dawley rats were investigated. This study confirms other findings that Sprague-Dawley rats fed an HFD for a long period of time, develop obesity, with a predominant accumulation of visceral fat as compared to rats on normal rat chow [30]. Usually, researchers opt for a higher percentage of fat (60%) so as to induce obesity in as little as 6 weeks [9]. If extrapolated to the human diet, a 60% fat content might not be realistic. Therefore the present study which uses a HFD with 36% of total energy from coconut fat is in line with recent studies which used HFDs with a reduced fat content (36–40%) with a longer time to induce obesity [31]. Rats on the HFD showed a 172% increase of their initial body weight after 12 weeks, while supplementation with MLE60 reduced weight gain. Rats receiving 150 and 350 mg/kg of MLE60 together with the HFD, showed no significant difference (*p* < 0.05) in weight gain as compared to rats receiving the ND (Figure 2). In terms of food intake, rats on the ND (3.27 kcal/g) consumed more in terms of volume as compared to the HFD but have lesser weight gain, thus confirming the higher calorie intake in the HFD group (4.39 kcal/g). The supplementation of MLE60 did not affect food intake in the HFD group, indicating that appetite suppression might not be the main anti-obesity mechanism involved (Table 4). Specifically to *M. citrifolia*, Mandukhail et al. reported prevention in weight gain in rats fed a HFD to induce hyperlipidaemia [14]. However, a reduction of food intake of rats fed extracts of the fruits and leaves were also reported. This can be attributed to the high dosage used (1000 mg/kg body weight), which could have affected the palatability of the diet.

**Table 3.** Chemical shifts assignments of characteristics ^1^H NMR signals of some metabolites in *M. citrifolia* extract (MLE60).

Metabolite	^1^H NMR characteristics
β-Glucose	4.59 (*d, J = *7.5 Hz)
α-Glucose	5.19 (*d, J = *3.5 Hz)
Sucrose	5.41 (*d, J =* 4 Hz)
Rutin	6.22 (*d, J = *2.0 Hz)
	6.39 (*d, J = *2.0 Hz)
	7.47 (*d, J = *2.0 Hz)
	6.90 (*d, J = *8.5 Hz)
	7.56 (*dd, J = *2.0, 8.5)
	anomeric proton glucosyl 4.95 (*d, J *= 8.0)
	rhamnosyl 4.58 (*d, J *= 1.0)
Kaempherol-3-*O*-α-l-rhamnopyranosyl-[1–6]-β-d-glucopyranoside	8.07 (*d, J =* 8.8 Hz)
	6.90 (*d, J = *8.8 Hz)
	6.40 (*d, J = *1.8 Hz)
	6.21 (*d, J =* 1.8 Hz)
	5.15 (*d, J = *7.6 Hz)
	4.54 (*s)*
	3.20–3.90 (*m)*
	1.14 (*d, J =* 5.8 Hz)
Catechin	6.47 (*d, J *= 2.0 Hz)
	4.57 (*d, J *= 7.5 Hz)
	6.50 (*d, J =* 2.0 Hz)
	2.56 (*dd, J *= 7.5, 16 Hz)
	3.98 (*m*)

*s*: singlet, *d*: doublet, *t*: triplet, *dd*: doublet of doublets, *m*: multiplet

The difference in weight gain among the different groups is clearly demonstrated by the percentage of visceral fat depot, where rats on the HFD plus MLE60 had significantly (*p* < 0.05) reduced percentage of visceral fat (3.45–4.04%) as compared to rats on the HFD only (6.08%) (Table 4). The results of this study are quite similar to those reported by Yamamoto et al. whereby weight gain was observed in lean rats fed a HFD with and without the administration of CT-II extract [32]. Lean rats receiving 2.5% of the extract had a suppressed weight gain during the 8 weeks of treatment, with a reduced percentage of parametrial fat. This effect was most likely to be mediated by an inhibition of digestive lipases. Similarly, the anti-obesity effects of *G. thundergii* extract in HFD-induced obese mice was investigated. Supplementation with the extracts for 6.5 weeks decreased body weight, adipose tissue mass, with a general amelioration in the serum lipid, leptin, and adiponectin profile [33].

Based on previous studies [34], it is postulated that MLE60 could have an anti-obesity effect through the inhibition of metabolic and digestive lipases, mimicking a reduced calorie intake. The faecal fat content was assessed as an indication of enzymes inhibition. The faecal fat content of rats in the HFD+150 (14.72, 11.37%) and HFD+350 (16.073, 19.58%) seem to support this hypothesis, as in both week 6 and week 12 of the study, rats receiving MLE60 had higher faecal fat content as compared to the control (Table 4). This suggests an inhibition of lipase by MLE60 *in vivo*. This is in line with previous studies showing that anti-obesity agents such as green tea increase energy content of faces in rats [35] and catechins from green tea showed increased faecal excretion of energy nutrients through the inhibition of digestive enzymes [36]. Potent lipase inhibitor, CT-II from Nomame Herba, resulted in significantly higher steatorrhea as compared to rats in the HFD group [32]. There was no indication of diarrhoea and abnormal lipid biochemistry in the subjects, suggesting that MLE60 has lesser side effects as opposed to Orlistat®, where abdominal discomfort and diarrhoea has been reported in humans [37].

Rutin and kaempherol were among the predominant flavonoids present in MLE60. These compounds together with other flavonoids like quercetin have been identified in the leaves of *M. citrifolia* previously [27]. Rutin was found to be the most potent in suppressing 3T3-L1 cell differentiation, in a dose dependent manner. At the induction stage of adipogenesis, rutin suppressed the activity of glycerol-3-phosphate dehydrogenase and activity of adipogenic factors such as peroxisome proliferator-activated receptor-γ (PPAR-γ) and CCAAT/enhancer binding protein-α (C/EBPα) in 3T3-L1 cells. *In vivo*, supplementation of rutin (25 mg and 50 mg/kg body weight) for 4 weeks in C57BL/6 mice fed a HFD resulted in reduced weight gain as compared to mice fed the HFD only. The anti-obesity effect of rutin was most likely to be mediated by the down regulation of certain adipogenic transcription factors as reflected by the down regulation of PPAR-γ and C/EBPα mRNA [38]. In obese Wistar rats, a 8 weeks intervention with 50 and 100 mg rutin /kg body weight, reduced body weight and adipose tissue, with amelioration in the serum lipid profiles, insulin, leptin, TG, cholesterol level, and oxidative profiles, confirming the protective effect of rutin in HFD induced dyslipidaemia, hepatosteatosis, and oxidative stress [29].

### Effect of MLE60 on plasma biochemistry

Apart from increased weight and fat accumulation, obesity is associated with other physiological disruptions, which is reflected by changes in the plasma biochemistry. A number of plasma components were measured in the treated and untreated HFD group and compared with the plasma profiles of rats fed the ND.

In general, MLE60 positively influenced the lipid profiles in the treatment group receiving the HFD (Table 4). The effect of purified HFD on TC is inconsistent though in most cases, hypercholesterolemia is not induced by diet only but with the addition of cholesterol. Only in the case of a fish fat based HFD, hypocholesterolaemia is induced [30]. A reduction in LDL level was recorded in both treated groups. Phenolic compounds such as rutin have been reported to positively modulate cholesterol metabolism. Rats receiving 1 g of cholesterol/kg for 5 weeks had lower plasma lipids and hepatic cholesterol with supplementation of rutin (1 g/kg body weight). The cholesterol lowering potential was mediated through the inhibition of HMG-CoA reductase and ACAT activity and an increased faecal sterol excretion. The study also reported the protective effect of rutin and tannic acid against oxidative stress [39].

Both a low dose (50 mg/kg) and a high dose of rutin (100 mg/kg) in rats fed a HFD containing 40% fat from beef tallow resulted in an improvement in the lipids profile in a dose dependent manner. LDL cholesterol and TC levels were reduced in the treated groups, without any changes in the HDL [29].

The HFD only group had significant higher plasma insulin levels (0.72 ± 0.07 μg/L) as compared to rats receiving the ND only (0.215 ± 0.01 μg/L) (Table 3). Administration of MLE60 improved plasma insulin dose dependently, with rats receiving 350 mg/kg body weight having the most improved profile (0.34 ± 0.05 μg/L), followed by rats receiving 150 mg/kg body weight (0.46 ± 0.06 μg/L). Various studies report an increase in plasma glucose and insulin levels with the administration of a HFD [40]. The insulin level was found to be increased 3.6 folds in the group receiving the HFD as compared to rats on the ND. Groups receiving the HFD together with different doses of MLE had improved insulin level, though not same as the normal rats. This might have been the causative effect of decreased visceral fats in the groups receiving the HFD together MLE60, whereby the visceral fat though reduced, was not comparable to the rats receiving the ND. Insulin insensitivity has been long been associated with an increase in visceral adiposity. People suffering from visceral obesity have a higher chance of having impaired insulin sensitivity and targeted weight loss in the visceral area can ameliorate insulin sensitivity [41].

Similar trend has been reported by Kishino et al. whereby insulin level was increased four times in female mice receiving a HFD as compared to mice on the ND [42]. Feeding of a mixture of *S. reticulata* aqueous extract and cyclodextrin (0.2% and 0.5%) resulted in an improvement of the insulin levels. This effect was attributed to the secondary modulation of metabolism associated with reduced visceral fat.

Leptin plays a predominant role in regulating food intake and energy expenditure, depending on the body fat content. People having a higher BMI have high serum and plasma leptin concentration. As opposed to insulin sensitivity, which improves with decrease in visceral fat, leptin can exert long acting effect to reduce adiposity [43].

In this study, the level of leptin was markedly reduced in the group receiving the HFD together with MLE60: the highest administered dosage of 350 mg/kg resulted in a twofold reduction (935.6 pg/mL) comparable to the control normal group (535.3 pg/mL) (Table 4). Other plant extracts associated with anti-obesity effects have improved plasma leptin profiles in subjects fed an HFD. Administration of various dosages of Yerba Mate (*I. paraguariensis*) in C57BL/6J mice resulted in a three times reduction of leptin concentration as compared to the control group. The extract showed both anti-obesity and anti-diabetic effect *in vivo* [9].

Circulating leptin levels is proportional to general adiposity and reduced adipose fat can result in decrease leptin concentrations [44]. Based on the fact that leptin in an appetite regulating hormone, it is expected to affect the appetite of rats in the groups having improved leptin levels. However, no changes were observed in food intake of rats within the groups receiving the same diets. Hayamizu et al. reported similar findings for the leptin lowering effect of *G. cambogia* extracts in mice fed a HFD [45]. In their study also, there was a direct correlation between leptin concentration and adiposity. It is proposed that this effect was through a leptin like activity of the extract, based on the relation between insulin and leptin.

Results from the present study also tend to show similar trends in the plasma concentration of leptin and insulin. The presence of leptin receptors on pancreatic β-cells has suggested possible involvement of leptin in insulin secretion [46]. This was further confirmed when a physiological increase in plasma leptin inhibited insulin secretion *in vivo*, in a dose dependent manner, independent of the cAMP phosphodiesterase 3 inhibitor [47].

Adiponectin, a hormone secreted by adipocytes has been shown to improve insulin resistance by decreasing the triglycerides content in muscles and liver of obese rodents. Its insulin sensitising properties include the suppression of hepatic glucose production and enhancing glucose uptake by the skeletal muscles [48]. In human subjects, plasma concentration of adiponectin is correlated with insulin sensitivity [49].

Similar to the effects in humans, the adiponectin levels in leptin-deficient, spontaneously obese mice were lower in obese mice than in lean mice. An extract of green tea did not ameliorate the adiponectin level in the HFD group [50]. On the contrary, Kishino et al. reported a significant decrease in the adiponectin levels of rats receiving a HFD with an extract of *K. himbutu* as compared to rats receiving the HFD only [42]. Some studies reported adiponectin levels to be unchanged in mice fed a HFD for 12 weeks in both the control and the treatment group [31]. This has been comprehensively studied by Barnea et al., who reported to the unchanged levels of adiponectin in mice fed an HFD, by no yet elucidated mechanisms of protection in rodents against decreased level of adiponectin [51].

Findings from this study showed no significant difference in adiponectin levels in all groups (13.39–18.12 ng/mL), though the level of adiponectin in obese rats was the lowest (13.39 ng/mL). Inter-individual variability might be one of the reasons of non-significance, since the trends in leptin and insulin levels suggest otherwise.

Rats on the HFD only had the lowest plasma ghrelin level (30.075 ng/mL), which was restored to the normal levels (61.311 ng/mL) by supplementation of MLE60 at the dosage of 150 mg/kg (66.613 ng/mL). A higher dose of MLE60 at 250 mg/kg did not improve plasma ghrelin levels. Ghrelin levels are generally decreased in human obesity and at meal times. It has also been proposed that ghrelin receptor can be good targets for anti-obesity drug, through appetite suppression [52]. In this study, the levels of ghrelin in HFD fed rats treated with MLE60 were improved even though no significant difference in food intake. Similarly, a water-alcohol naringin rich extract of *C. grandis* improved ghrelin levels in obese Zucker rats [53].

The overall kidney function is measured by the glomerular filtration rate (GFR), which cannot be assessed directly. Filtration markers such as creatinine and urea are used as renal function indicators, clinically [54]. In this study, the creatinine levels among the various groups were not significantly different, in both rats fed the HFD (53.00 μmol/L) or the ND (54.75 μmol/L). Results are consistent with various studies reporting unchanged levels of creatinine in obese subjects as there is no change is muscle mass [55].

The blood urea nitrogen (BUN) was also measured as indicator of renal function. Rats fed a HFD and HFD+150 had significantly lower urea content as compared to rats fed the ND. The higher dosage of MLE60 (350 mg/kg) improved the urea levels in the treated group. Consistent with findings from this study, blood urea level has been previously reported to be decreased in obese rats, irrespective of whether being fed a palatable cafeteria diet or a standard chow. This suggests that decreased urea level was related to the condition of obesity rather than the diet [56].

In the current study, four markers of liver function were measured (GGT, ALP, AST, and ALT). Significant changes were only observed in the GGT and ALP levels. Obese rats had significantly higher ALP (149.25 U/L) and GGT (6.0 U/L) levels than their lean counterparts, with 70.75 and 1.0 U/L, respectively. Administration of MLE60 at various doses improved the GGT and ALP levels in rats fed a HFD and did not cause significant changes in rats fed a ND.

Green tea extract reduced hepatic liver injury, as reflected by decreased levels of ALT and AST. This effect was attributed to the lipid lowering potential of the green tea extract [50]. In a similar study, serum AST, ALP and ALT were increased in rats fed a HFD. Groups supplemented with raspberry ketone had lower levels of enzymes. The attenuation of steatohepatitis was attributed to decreased liver inflammation, improvement of dyslipidaemia, and increased sensitivity to leptin and insulin [57].

As a preliminary indicator of toxicity, the weight of all organs was recorded upon sacrifice. No significant findings were recorded, especially in the treated group, suggesting that MLE60 was not toxic at the administered dosages (Table 5). Moreover, *M. citrifolia* is well known for its history of safe use and no perceived toxicity across the world, except some isolated cases due to the extreme consumption of noni juice [16]. Testing of *M. citrifolia* did not show any sign of toxicity in rats [58] and LD_50_ values of 7500 mg/kg and 3500 mg/kg were determined for the aqueous and ethanol extracts, respectively [59]. In this study, a maximum dosage of 350 mg/kg of ethanolic extract was used, an equivalent of 1 g of powdered leaves. However, more profound toxicology studies are required in the event that MLE60 is further advised to be used for preventing weight gain.

**Table 4.** The daily food intake, visceral fat content, faecal fat excretion, plasma lipids, glucose, insulin, leptin, adiponectin, ghrelin, and toxicity markers levels in rats fed a normal diet (ND) or a high fat diet (HFD) after 12 weeks of supplementation with or without MLE60 at 150 mg/kg or 350 mg/kg body weight.

	HFD	HFD+150	HFD+350	ND	ND+150	ND+350
Daily food intake (g/rat/day)	16.45 ± 2.60^c^	15.38 ± 4.10^c^	14.54 ± 2.60^c^	21.98 ± 1.80^a^	18.77 ± 2.00^b^	20.28 ± 1.70 ^ab^
Visceral fat (%)	6.98 ± 0.63^c^	4.04 ± 0.80^b^	4.45 ± 0.96^b^	2.07 ± 0.58^a^	1.40 ± 0.49^a^	1.97 ± 0.28^a^
Faecal fat content (%) Middle (Week 6) Final (Week 12)	4.40 ± 0.82^aA^	14.73 ± 1.68^aB^	16.07 ± 2.81^aB^	3.28 ± 0.34^aA^	2.80 ± 0.27^aA^	5.07 ± 0.34^aA^
	5.34 ± 0.62^aB^	11.37 ± 0.56^aB^	19.58 ± 3.36 ^aA^	6.43 ± 1.76^aB^	10.67 ± 1.04^bB^	10.42 ± 0.76^bB^
Total cholesterol (mmol/L)	1.28 ± 0.05^c^	0.86 ± 0.05^a^	0.95 ± 0.19^a^	1.36 ± 0.17^b^	1.26 ± 0.14^bc^	1.35 ± 0.05^bc^
HDL (mmol/L)	0.63 ± 0.10^b^	0.65 ± 0.08^b^	0.72 ± 0.05^ab^	1.03 ± 0.16^a^	0.90 ± 0.11^a^	1.00 ± 0.04^a^
LDL (mmol/L)	0.25 ± 0.04^b^	0.14 ± 0.02^a^	0.13 ± 0.02^a^	0.29 ± 0.05^b^	0.30 ± 0.04^b^	0.30 ± 0.03^b^
Triglycerides (mmol/L)	1.01 ± 0.05^c^	0.36 ± 0.11^ab^	0.51 ± 0.06^b^	0.25 ± 0.06^a^	0.29 ± 0.04^a^	0.24 ± 0.02^a^
Glucose (mmol/L)	6.48 ± 0.76^b^	6.20 ± 1.19^b^	6.15 ± 0.81^b^	4.80 ± 0.16^a^	5.03 ± 0.60^ab^	5.08 ± 0.01^ab^
Insulin (μg/L)	0.72 ± 0.08^d^	0.46 ± 0.06^c^	0.34 ± 0.05^b^	0.22 ± 0.01^a^	0.18 ± 0.01^a^	0.14 ± 0.01^a^
Leptin (pg/mL)	1948.9 ± 46.7^d^	1627.3 ± 216.3^c^	935.6 ± 140.3^b^	535.3 ± 12.9^a^	556.7 ± 18.6^a^	377.8 ± 40.2^a^
Adiponectin (ng/mL)	13.39 ± 2.00^a^	13.99 ± 1.71^a^	15.39 ± 2.81^a^	16.39 ± 2.28^a^	18.13 ± 5.61^a^	14.09 ± 0.45^a^
Ghrelin (ng/mL)	30.08 ± 7.78^b^	61.61 ± 8.84^a^	30.40 ± 8.61^b^	61.31 ± 1.07^a^	52.68 ± 8.42^a^	61.32 ± 1.53^a^
Creatinine (μmol/L)	53.00 ± 2.94^a^	53.00 ± 3.56^a^	54.00 ± 3.56^a^	54.75 ± 2.22^a^	55.25 ± 2.36^a^	60.75 ± 1.89^a^
Urea (mmol/L)	3.36 ± 0.05^a^	3.16 ± 0.45^a^	5.05 ± 0.17^b^	5.70 ± 0.22^c^	6.45 ± 0.46^c^	6.10 ± 0.96^bc^
GGT (U/L)	6.00 ± 2.16^c^	2.00 ± 1.15^ab^	3.50 ± 0.57^b^	1.00 ± 0.00^a^	1.00 ± 0.00^a^	1.00 ± 0.00^a^
AST (U/L)	94.63 ± 1.90^a^	92.55 ± 2.70^a^	88.80 ± 2.89^a^	90.35 ± 6.27^a^	99.95 ± 19.91^a^	104.13 ± 18.09^a^
ALT (U/L)	32.15 ± 3.28^a^	32.53 ± 5.45^a^	32.63 ± 5.24^a^	34.48 ± 2.29^a^	44.23 ± 5.49^b^	37.08 ± 2.58^a^
ALP (U/L)	149.25 ± 15.52^c^	114.50 ± 17.60^b^	114.00 ± 5.77^b^	70.75 ± 5.32^a^	90.25 ± 6.90^ab^	97.00 ± 14.51^ab^

Different small letters indicate significance difference (*p* < 0.05) between different groups and difference in big letters indicate significance difference in the same group at different time points as shown by the analysis of variance (ANOVA) using MINITAB VERSION 14.

**Table 5.** Weight of organs (g).

Group	Liver	Kidney	Heart	Lung	Testis
HFD	9.05 ± 1.23^a^	2.27 ± 0.26^a^	1.24 ± 0.8^a^	1.46 ± 0.28^a^	1.24 ± 0.41^b^
HFD+350	8.06 ± 1.46^ab^	2.04 ± 0.16^ab^	1.22 ± 0.14^a^	1.49 ± 0.25^a^	1.48 ± 0.17^ab^
HFD+150	8.99 ± 0.85^ab^	2.14 ± 0.2^ab^	1.23 ± 0.14^a^	2.01 ± 0.87^a^	1.48 ± 0.14^ab^
ND	9.11 ± 1.82^ab^	2.44 ± 0.42^a^	1.21 ± 0.20^a^	1.49 ± 0.30^a^	1.69 ± 0.31^a^
ND+150	6.88 ± 0.42^b^	1.83 ± 0.08^b^	0.94 ± 0.15^a^	1.38 ± 0.13^a^	1.47 ± 0.13^ab^
ND+350	7.63 ± 0.77^ab^	2.26 ± 0.28^ab^	1.20 ± 0.08^a^	1.50 ± 0.05^a^	1.70 ± 0.08^a^

Different letters indicate significance difference (*p* < 0.05) of the same organ between different groups as shown by the analysis of variance (ANOVA) using MINITAB version 14.

## Conclusion

Rats receiving the HFD together with MLE60 had reduced weight gain as compared to rats receiving the HFD only. The weight gain in the treated groups was not significantly different to weight gain by rats fed a ND. Daily food intake did not differ between the treated and control groups, therefore eliminating appetite suppression as one of the potential anti-obesity mechanism. Increased faecal fat excretion at week 6 and week 12 reinforces the initial hypothesis of lipase inhibition by MLE60. The plasma lipid profiles were also improved, with a marked decrease in plasma TG and LDL levels. Other pro-obesity related factors such as plasma insulin and leptin were significantly improved, suggesting that in addition to its lipase inhibitory effects; MLE60 positively modulates adipocytic mechanisms through a leptin like activity, to exhibit anti-obesity properties. Supplementation of the extract at the prescribed dosages did not adversely affect liver and kidney toxicity markers. No abnormal bowel activities (no oily stools, no diarrhoea) were recorded in the treated group, as it is the case with the commonly prescribed anti-obesity drug, pancreatic lipase inhibitor, Orlistat® at the required dosage of 120 mg, three times a day. However, more advanced toxicity studies are required for conclusive results.

MLE60 shows promise as a natural and safe anti-obesity agent and warrants further investigation. In this study, the effect of the extract was assessed in lean rats fed a HFD, to mimic the current trend of high caloric environment and possible prevention of obesity in lean subjects. It would be interesting to study the effect of the same extract on HFD induced obese models, to verify whether MLE60 can be curative, as well as preventive.
